# Radial, renal and craniofacial anomalies: Baller-Gerold syndrome

**DOI:** 10.4103/0970-0358.41118

**Published:** 2008

**Authors:** Jyotsna Murthy, Ramesh Babu, Padmasani Venkat Ramanan

**Affiliations:** Department of Plastic Surgery, Director of Cleft and Craniofacial Center, Sri Ramachandra Medical College and Research Institute, Porur, Chennai, Tamil Nadu, India; 1 Consultant Pediatric Urologist, Sri Ramachandra Medical College and Research Institute, Porur, Chennai, Tamil Nadu, India; 2 Department of Pediatrics, Sri Ramachandra Medical College and Research Institute, Porur, Chennai, Tamil Nadu, India

**Keywords:** Baller-Gerold syndrome, craniosynostosis, crossed ectopia, ectopic kidneys, microcephaly, radial agenesis, radial club hand, reflux, renal agenesis, vesico ureteric reflux

## Abstract

The Baller-Gerold syndrome is a rare syndrome with very few cases published in literature. Craniosynostosis and radial aplasia are striking features, easy to diagnose. However, there are many differential diagnoses. Often, the question raised is whether the Baller-Gerald syndrome is a distinct entity. We report a patient with findings of craniosynostosis and radial aplasia consistent with the diagnosis of the Baller-Gerold syndrome. Genotypic heterogeneity could possibly underlie the phenotypic variability exhibited by these cases.

## INTRODUCTION

Baller[1] described a female with oxycephaly and absent radius whose parents were third cousins. Gerold[2] described a brother and sister, aged 16 years and two days, respectively with tower skull, radial aplasia and small ulna. The term Baller-Gerold syndrome was coined by Cohen[3] to designate the phenotype of craniostysnostosis and radial dysplasia. The Baller-Gerold Syndrome is a rare genetic disorder of which only 22 cases have been reported.[4] Although initially only craniosynostosis and preaxial upper limb defects were included,[5,6] the phenotype outlined in later reviews[7–9] included growth deficiency, sudden infant death syndrome, a peculiar facial appearance with malformed and low set ears, a prominent nasal bridge, epicanthus, micrognathia, cleft palate, bifid uvula, high arched palate and a prominent mandible. Other systems involved are cardiac (ventricular septal defect (VSD), patent ductus arteriosus, supravalvular aortic stenosis), renal (ectopia, agenesis), anal (imperforated anus, anterior anus, perineal fistulae), nervous system (polymicrogria, hydrocephalus, agenesis of corpus callosum or seizure disorder) and skeletal (joint limitation, rib fusion, flat vertebrae and absent middle phalanx). They are divided into two groups - those with craniosynostosis and radial defect alone and those with additional malformation. Children with additional malformations are particularly affected by growth and mental retardation. We report here a patient with the finding of craniosynostosis and radial aplasia consistent with the diagnosis of the Baller-Gerold syndrome.

## CASE HISTORY

**Figure 1:**
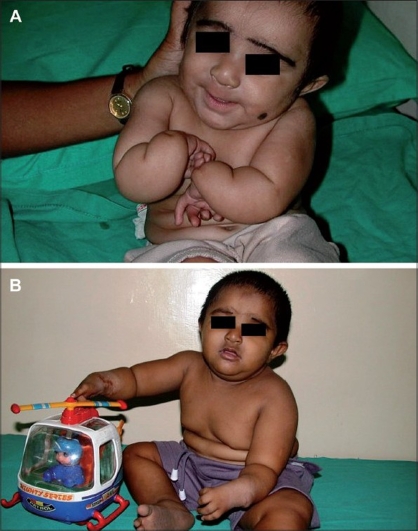
Bilateral radical club hand (A) before and (B) after surgery

**Figure 2:**
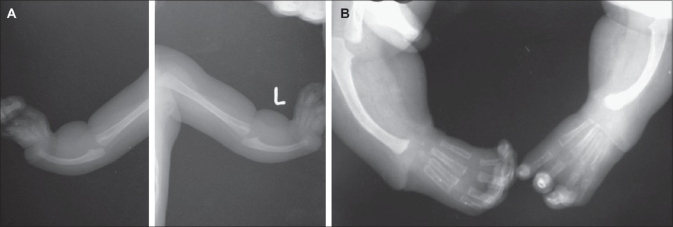
X-ray of bilateral Club hand (A) before and (B) after surgery

**Figure 3:**
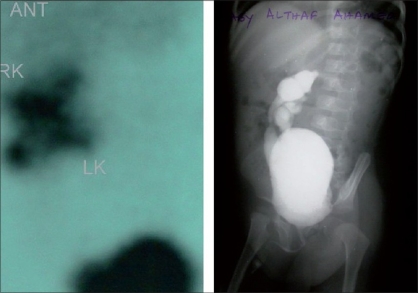
Renal anomalies: Both kidneys on the same side (DMSA) and hydroureter (MCU)

A male child born at full term with a birth weight of only 2.2 kg was noted to have facial and limb abnormalities at birth. The parents were first cousins (consanguinous marriage) but did not have any abnormalities or family history of disorders. He had a typical face with low hairline, small eyes, a prominent nasal bridge and prominent ears. Microcephaly (head circumference = 29 cm) was noticed at birth. The calvarium was of normal shape and fontanels were closed in the normal way. The child had bilateral radial dysplasia with complete absence of radius and thumbs [Figures 1, 2]. There were no other skeletal abnormalities. Clinical examinations of cardiac, gastrointestinal and central nervous systems were normal at birth. The echocardiogram was also within normal limits. Routine chromosome analysis showed a normal karyotype and was negative for Fanconi anemia. The development was normal but the child was at the lowest percentile for height, weight and head circumference for his age and had delayed eruption of teeth. At the age of fifteen months, both radial club hands were corrected and hands were centralized on the ulna with tendon transfer. The child suffered repeated urinary infections when he was two years old. A renal scan showed that both kidneys were on the right side, fused one over the other with hydroureteronephrosis. A dimercaptosuccinic acid (DMSA) scan confirmed equal function and no scarring in the crossed, fused ectopic kidney. Micturition cystourethrography (MCU) revealed bilateral grade IV reflux with the left ureter crossing across to the right-sided ectopic kidney [Figure 3]. Blood biochemistry revealed slightly elevated creatinine, normal urea and electrolytes. Bilateral extravesical reimplantation of the ureters was performed. The postoperative period was uneventful and follow-up at three months with ultrasound and indirect nuclear cystogram revealed good kidney function and no vesico-ureteric reflux. His general assessment at the age of four years was satisfactory except for delayed speech and language.

## DISCUSSION

The fifth week of embryonic development is crucial to the development of many organs including the heart, kidneys and limbs. Any insult during this stage can result in the coexistence of multiple abnormalities. The Baller-Gerold Syndrome is a genetically determined growth deficiency syndrome with premature craniosynostosis, hypoplasia or the absence of a radius, delayed psychomotor development and variable craniofacial, cardiac, renal and skeletal defects.[10,11] Due to premature fusion of the fibrous joints between certain bones in the skull (craniosynostosis), the head may appear unusually short, wide or relatively triangular in shape. In addition to underdevelopment or absence of the radius, the ulna may be unusually short and curved and the thumbs may be underdeveloped or absent. In some cases, additional physical abnormalities and/or mental retardation may also be present. The Baller-Gerold Syndrome is thought to be inherited as an autosomal recessive trait; sudden infant death is common.

The patient described in this report had craniofacial abnormalities, radial dysplasia and renal abnormalities without any proximal skeletal involvement and haemopoeitic abnormalities. A radial dysplasia is generally corrected in two stages: the first stage involves centralization of hand on the ulna with tend transfer while the second stage is pollicisation depending on the progress of the child. Crossed ectopia and renal agenesis *per se* do not require any intervention. However, reflux or obstruction in the presence of such anatomical abnormalities are unlikely to resolve and will need surgical correction.

The presence of craniofacial abnormality and radial club hands should alert one to look into the other features of this syndrome. A phenotypic overlapping of Baller-Gerold and Robers-SC syndrome has been noted.[12] However, Roberts-SC syndrome is associated with anomalies of the proximal bone and with deformities like phocomelia. Thus, the differential diagnoses to be considered include: Fanconi anaemia, Holt Oram Syndrome, Roberts Syndrome, VACTERAL association (vertebral defects, anorectal atresia, cardiac defects, tracheal oesophageal fistula, eosophageal atresia, renal abnormalities and limb abnormalities) and TAR (thrombocytopenia with absent radius) syndrome.[13] A mutation in TWIST, a basic helix-loop-helix transcription factor has been identified in patients with the Baller-Gerold Syndrome.[9,14] Molecular investigations will help to delineate the similarities or differences between presumed cases of the Baller-Gerold syndrome and other overlapping syndromes.
